# Associations of Baseline Cognitive and Psychosocial Function with Clinical Outcomes After Autologous Stem Cell Transplantation in a Multiple Myeloma Sample

**DOI:** 10.3390/cancers18203264

**Published:** 2026-10-10

**Authors:** Maria Rueda-Lara, Krishna V. Komanduri, Denise Pereira, Lazaros Lekakis, Trent Peng Wang, Antonio M. Jimenez, Amer Beitinjaneh, Noa G. Holtzman, Damian J. Green, Mark Goodman, Cara L. Benjamin, Philip D. Harvey, Brittany J. Wright

**Affiliations:** 1 Department of Psychiatry and Behavioral Sciences, Miller School of Medicine and Sylvester Comprehensive Cancer Center, Miami, FL 33136, USA; brittanywright33@med.miami.edu; 2 Department of Medicine, Division of Hematology and Oncology, Helen Diller Family Comprehensive Cancer Center, San Francisco, CA 94143, USA; krishna.komanduri@ucsf.edu; 3 Department of Medicine, Division of Transplantation and Cellular Therapy, Miller School of Medicine and Sylvester Comprehensive Cancer Center, Miami, FL 33136, USA; dpereira2@med.miami.edu (D.P.); llekakis@med.miami.edu (L.L.); trentwang@med.miami.edu (T.P.W.); amjimenez@med.miami.edu (A.M.J.); abeitinjaneh@miami.edu (A.B.); ngh45@med.miami.edu (N.G.H.); damiangreen@miami.edu (D.J.G.); mgoodman@med.miami.edu (M.G.); c.benjamin3@med.miami.edu (C.L.B.); 4 Department of Psychiatry and Behavioral Sciences, Division of Psychology, Miller School of Medicine, Miami, FL 33136, USA; pharvey@med.miami.edu

**Keywords:** anxiety, memory, length of stay

## Abstract

**Background/Objectives:** Autologous stem cell transplantation (ASCT) remains a standard frontline treatment for eligible patients with multiple myeloma and is associated with improved progression-free survival and sustained treatment responses. In addition to medical eligibility factors, pre-treatment psychosocial variables such as depression and anxiety as well as cognitive functioning may influence short- and long-term transplant outcomes. This study examined the relationship between pre-transplant psychosocial and cognitive functioning and clinical outcomes in patients with multiple myeloma undergoing ASCT. **Methods:** Participants (*n* = 245) undergoing a standardized protocol for ASCT in Miami, Florida received pre-transplant psychosocial and neuropsychological assessments. Patient-reported clinical variables and cognitive performance were related to transplant success and transplant recovery outcomes, including time to engraftment, length of hospital stay post-engraftment, and survival across four timepoints. **Results:** While most patients achieved engraftment within a similar timeframe, the duration of hospitalization following engraftment varied considerably. Only higher baseline trait anxiety was associated with longer post-engraftment hospital stays. In multivariate Cox regression analyses, post-engraftment length of stay was independently associated with long-term mortality when modeled as a continuous variable. In a secondary analysis using an empirically derived cutoff corresponding to stays longer than the 80th percentile (≥7 days post-engraftment), both prolonged post-engraftment hospitalization and poorer baseline delayed memory was independently associated with mortality. Descriptive survival analyses further showed that patients with prolonged post-engraftment hospitalizations had higher overall mortality and shorter survival than those with shorter post-engraftment hospital stays. **Conclusions:** Patients who demonstrated poorer delayed memory performance pre-transplant and prolonged hospital admission post-transplant may be most vulnerable to dying when examined at long-term follow-up. Pre-transplant psychiatric assessments provide an opportunity to identify and potentially implement supportive care interventions for patients at increased risk for cognitive impairment.

## Simple Summary

Autologous stem cell transplantation is an important treatment for eligible patients with multiple myeloma. Although medical factors are routinely considered when determining whether a patient is prepared for transplantation, less is known about how emotional well-being and cognitive functioning before treatment may influence recovery and long-term outcomes. In this study, we examined symptoms of anxiety and depression and cognitive abilities assessed before transplantation and related them to recovery after transplantation and long-term survival. We found that higher trait anxiety before transplantation was associated with a longer hospital stay after blood cell recovery, while longer hospitalization after recovery was associated with a greater risk of death during long-term follow-up. Poorer memory performance before transplantation was also associated with long-term mortality in a secondary analysis. These findings suggest that emotional and cognitive assessments before transplantation may provide useful information beyond traditional medical factors and may help researchers and clinical teams identify patients who could benefit from additional monitoring and supportive care. Further research is needed to determine whether interventions addressing these factors can improve recovery and long-term outcomes after transplantation.

## 1. Introduction

Multiple myeloma (MM) is the second most common hematologic malignancy in adults, caused by clonal plasma cell (CP) proliferation leading to organ damage. Treatment outcomes have significantly improved from a median overall survival of approximately 3 years with conventional chemotherapy prior to 2000, to over 10 years with modern combination regimens with proteasome inhibitors, immunomodulatory drugs, and anti-CD38 monoclonal antibodies [1,2]. Autologous stem cell transplantation (ASCT) remains a standard for eligible patients with newly diagnosed multiple myeloma, supported by category 1 evidence in the NCCN guidelines [3].

For planned inpatient ASCT, the most representative US national data suggest a mean total length of stay of approximately 13 days and a median of 16 days [4,5]. Contributors to prolonged length of stay (LOS) after autologous stem cell transplant (ASCT) for MM fall into three distinct but interrelated domains. Engraftment-dependent factors—infused CD34+ cell dose, cryopreservation quality, baseline cytopenias, and prior therapy effects such as daratumumab exposure—can affect the tempo of hematopoietic recovery and the core hospitalization period [6,7]. Non-engraftment complications, including febrile neutropenia, bacteremia, and regimen-related toxicity (e.g., gastrointestinal (GI) toxicity [mucositis and diarrhea from high-dose melphalan]) are post-engraftment complications; up to 27% of patients remain hospitalized despite successful engraftment [8,9,10]. Patient-level factors—comorbidity burden and baseline patient-reported psychosocial factors such as quality of life, depressive symptoms, and symptom burden—independently predict prolonged LOS and post-transplant resource utilization, even after adjustment for clinical and transplant-related variables [11,12].

This three-domain framework carries clinical significance: each element can necessitate a distinct intervention. Engraftment-related delays call for optimized graft collection and CD34+ dosing; post-engraftment complications call for targeted infection prevention and supportive care; and patient-level risk calls for integrated psychosocial screening and intervention.

Even after successful hematopoietic recovery, a substantial proportion of MM patients experience hospitalization prolonged beyond the point of engraftment. In a study of 100 ASCT recipients (including melphalan-conditioned MM patients), 27% experienced delayed discharge despite achieving engraftment, with a mean prolongation of 3 days (range 1–11) [10]. The dominant causes were entirely non-hematologic: GI toxicity accounted for 14.7% of post-engraftment delays in melphalan-conditioned patients (primarily diarrhea and mucositis from high-dose melphalan), while infectious complications accounted for 13%, and cardiotoxicity occurred in a small minority of cases [10]. This pattern was held at scale: in the CALM study of 3552 MM patients, bacterial infections (6.5 per 100 patient-years) and GI complications (4.7 per 100 patient-years) were the most common early events, with infectious complications occurring at twice the rate of non-infectious events within the first 100 days [13]. GI and neutropenic fever/infectious complications likewise dominated unscheduled admissions in outpatient ASCT models, where baseline creatinine > 1.5 mg/dL and HCT-CI > 2 independently predicted unscheduled hospitalization (HR 1.6, *p* = 0.001) [14].

Taken together, post-engraftment length of stay (LOS) in patients with MM likely reflects the convergence of treatment-related toxicity, infectious complications during immune reconstitution, and patient-level clinical and psychosocial vulnerabilities. Melphalan-related mucosal injury and GI toxicity, and infectious burden may directly delay medical recovery and discharge. However, emerging evidence suggests that pre-HSCT (Hematopoietic stem cell transplantation) patient characteristics may also influence the post-transplant course. Quality of life, symptom burden and depressive symptom severity, older age, greater comorbidity burden, psychosocial risk factors, and non-remission of disease have also been associated with prolonged LOS, re-admission, or delayed engraftment [11,15,16,17,18,19,20]. Collectively, these findings underscore the multifactorial nature of prolonged hospitalization following ASCT and highlight the potential importance of integrating clinical, functional, and psychosocial factors when identifying patients at risk for delayed recovery.

In this study, we examined time to engraftment and length of hospital stay after successful engraftment, as well as post-discharge survival over an up to 11-year post-transplant follow-up period among patients with MM undergoing ASCT at a single institution where all patients are admitted from start of conditioning. We related these three sequential outcomes to pre-transplant psychosocial risk factors, including mood and anxiety symptoms, perceived social support, smoking, and alcohol use, as well as performance-based assessments of cognitive functioning. Thus, we were able to separately predict all three outcomes from the pre-procedure data and relate time to engraftment to post-engraftment length of stay and examine the association of pre-procedure characteristics, time to engraftment, and hospital stay post-engraftment to survival in the short term and long term across four different time points. We hypothesized that baseline cognitive functioning and psychosocial variables would be associated with patients’ downstream clinical outcomes following ASCT and may serve as markers of vulnerability that ultimately correlate with survival post-transplant.

Our cohort is notable for its racial, ethnic, and linguistic diversity, including a large proportion of Hispanic immigrants, monolingual Spanish speakers, and Black patients from urban Miami-Dade County, many of whom experience significant psychosocial and healthcare access disparities.

## 2. Materials and Methods

### 2.1. Participants

All participants in this study were individuals with multiple myeloma eligible to receive an autologous HSCT. All participants underwent a routine psychosocial evaluation as a part of our institutional standard of care. No additional data was collected outside of these assessments. Each patient participated in a structured psychosocial interview conducted by one of three bilingual clinicians: a psychologist, a psychiatrist, or an advanced nurse practitioner with specialized training in neuropsychological assessment administration. Patients completed self-report measures and underwent a performance-based cognitive evaluation, conducted in either English or Spanish. Information about other elements of health status (e.g., alcohol use disorders) and demographic information were collected both from the psychosocial interviews and the participants’ hospital electronic health records.

### 2.2. Procedure

This study was conducted in Miami, FL, at the Sylvester Comprehensive Cancer Center, an NCI-designated cancer center. Each potential HSCT patient was identified by their medical team and if they met medical criteria to be eligible for the transplant, they were referred for a routine standard-of-care psychosocial evaluation required for all patients wherein participants completed the measures and clinical interview. All patients were admitted to the hospital for the ASCT, as our institution does not currently perform ASCT in the outpatient setting. Patients with multiple myeloma undergoing autologous stem cell transplantation (ASCT) at our institution were managed according to a standardized institutional transplant protocol. This protocol included established institutional procedures for conditioning therapy, stem cell infusion, antimicrobial prophylaxis, transfusion support, supportive care, monitoring of hematopoietic recovery, and criteria for hospital discharge, ensuring a consistent approach to transplant management across the study cohort. With respect to conditioning, all patients received melphalan 200 mg/m^2^ as the standard conditioning regimen according to the transplant center’s standard operating procedures (SOP), with dose adjustment based on renal function when clinically indicated. In addition, per institutional SOP, no patient proceeded to transplantation with a CD34+ cell dose below 2 × 10^6^ cells/kg. A retrospective review of electronic health records, including information about the nature and success of the transplant, was also performed under the approval of the University of Miami Institutional Review Board. All adult patients who underwent a psychosocial evaluation for HSCT by the psycho-oncology team of the Department of Psychiatry and Behavioral Sciences at Sylvester Comprehensive Cancer Center between 1 December 2011 and 30 June 2016 and received the HCST procedure were included.

### 2.3. Study Design

A retrospective records-based study included pre-transplant from all 245 patients, which were utilized in the analyses of pre-transplant psychological symptoms, cognitive functioning, and demographic characteristics. Only patients who subsequently underwent ASCT were included in the analyses of transplant-related outcomes, including time from transplantation to engraftment, post-transplant length of hospital stay, and post-procedure survival measured from time of transplant to 11 years post-transplant (range = 29–3757 days; median = 1159 days). Engraftment was defined according to the Center for International Blood and Marrow Transplant Research (CIBMTR) criteria. Neutrophil engraftment was defined as the first of three consecutive days with an absolute neutrophil count (ANC) ≥ 0.5 × 10^9^/L. Platelet engraftment was defined as the first of three consecutive platelet counts ≥20 × 10^9^/L obtained on different days, occurring at least 7 days after the last platelet transfusion [21]. Cognitive functioning was assessed using the Repeatable Battery for the Assessment of Neuropsychological Status (RBANS) [22] and Trail Making Test Parts A and B [23,24]. Certified Spanish translations of the assessment materials were used. Psychological and psychosocial symptoms were evaluated using the Beck Depression Inventory-II (BDI-II) [25], State-Trait Anxiety Inventory (STAI) [26,27], Alcohol Use Disorders Identification Test (AUDIT) [28], and Multidimensional Scale of Perceived Social Support (MSPSS) [29]. All these scales are scored such that higher scores reflect more symptoms or lower levels of social support. The RBANS is an abbreviated assessment focused on cognitive impairments that are commonly seen with aging in adults and includes assessments of immediate and delayed recall memory for verbal and non-verbal information as well as attention, visual-spatial, and language performance. Scores are standardized by age and are presented as age-corrected scaled scores with a normative mean of 100 and an SD of 15.

The Hematopoietic Cell Transplantation-Specific Comorbidity Index (HCT-CI) was collected by the medical staff as a standardized measure of pre-transplant comorbidity burden and an established predictor of non-relapse mortality and survival following HSCT [30]. This index measures 17 different organ categories or medical co-morbidities which are rated as present or absent, yielding a total score which is the commonly used predictor variable. HCT-CI score categorizes patients into one of the three risk categories: low, intermediate, and high. Additional clinical and behavioral variables included prior psychiatric history, tobacco use, hypertension, and diabetes.

### 2.4. Data Analysis

We examined the correlates of time to engraftment, length of stay in total and post-engraftment, and mortality. We used Pearson correlations to relate the continuous variables to each other, with the analyses focusing on variables that were collected prior to and after transplant. Thus, the interpretation of the results is informed by the fact that all outcome data were collected in sequence. For some analyses, survival was defined as a dichotomous variable, such that *t*-tests were performed to examine differences in the predictor variables, including both pre-transplant and the two lengths of stay variables. Finally, we examined length of post-transplant survival with Cox regression models as well as Kaplan–Meier life table statistics, using previously identified predictors of each of the three sequential transplant-related outcomes.

## 3. Results

Data on demographic characteristics of the participants and their scores on the mood, social support, and medical risk measures are presented in Table 1. Cognitive test performance is presented in Table 2, as are durations for time to engraftment, total and post-engraftment length of stay, and post-transplant survival rates.

**Figure 1:**
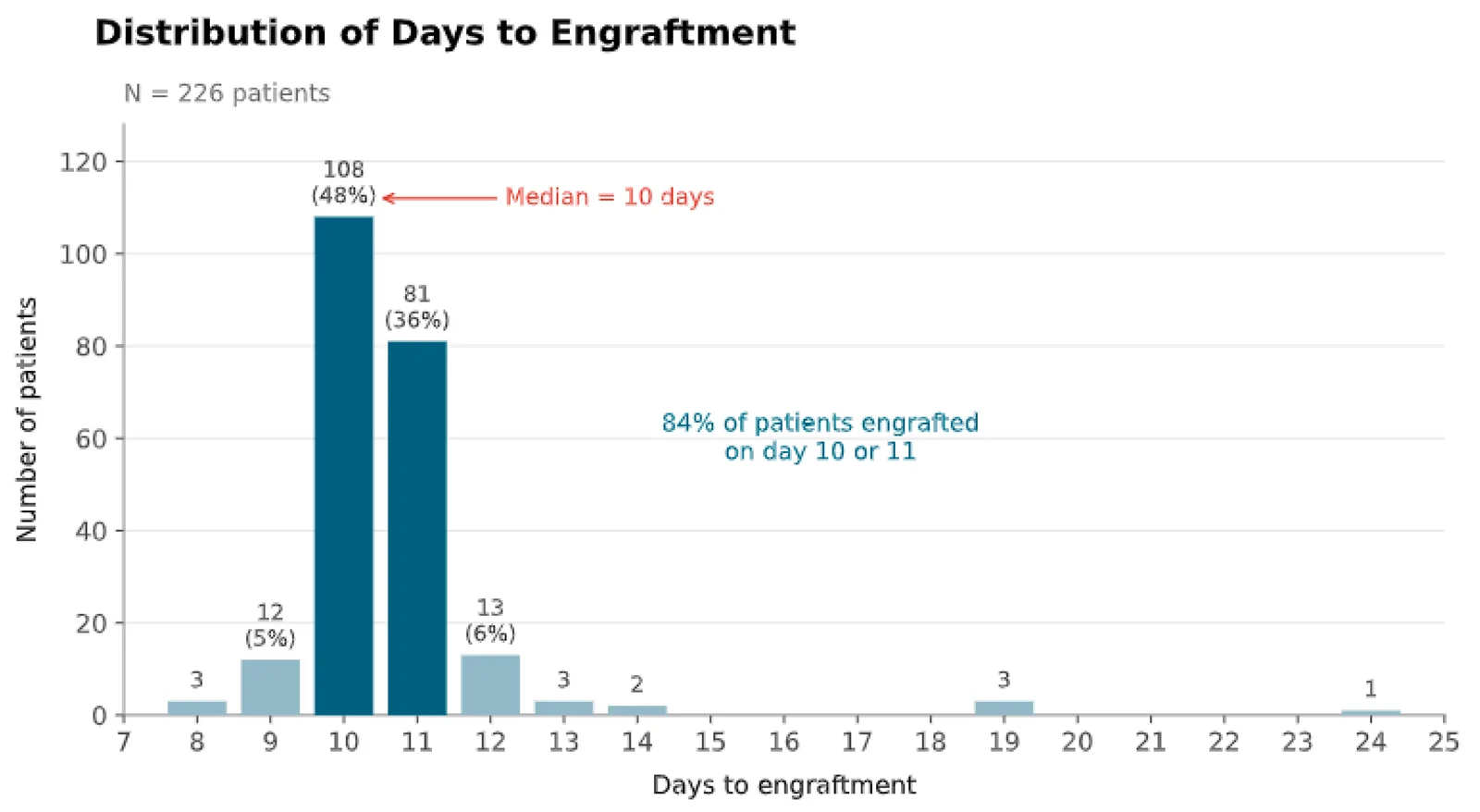
Distribution of number of days to engraftment.

**Figure 2:**
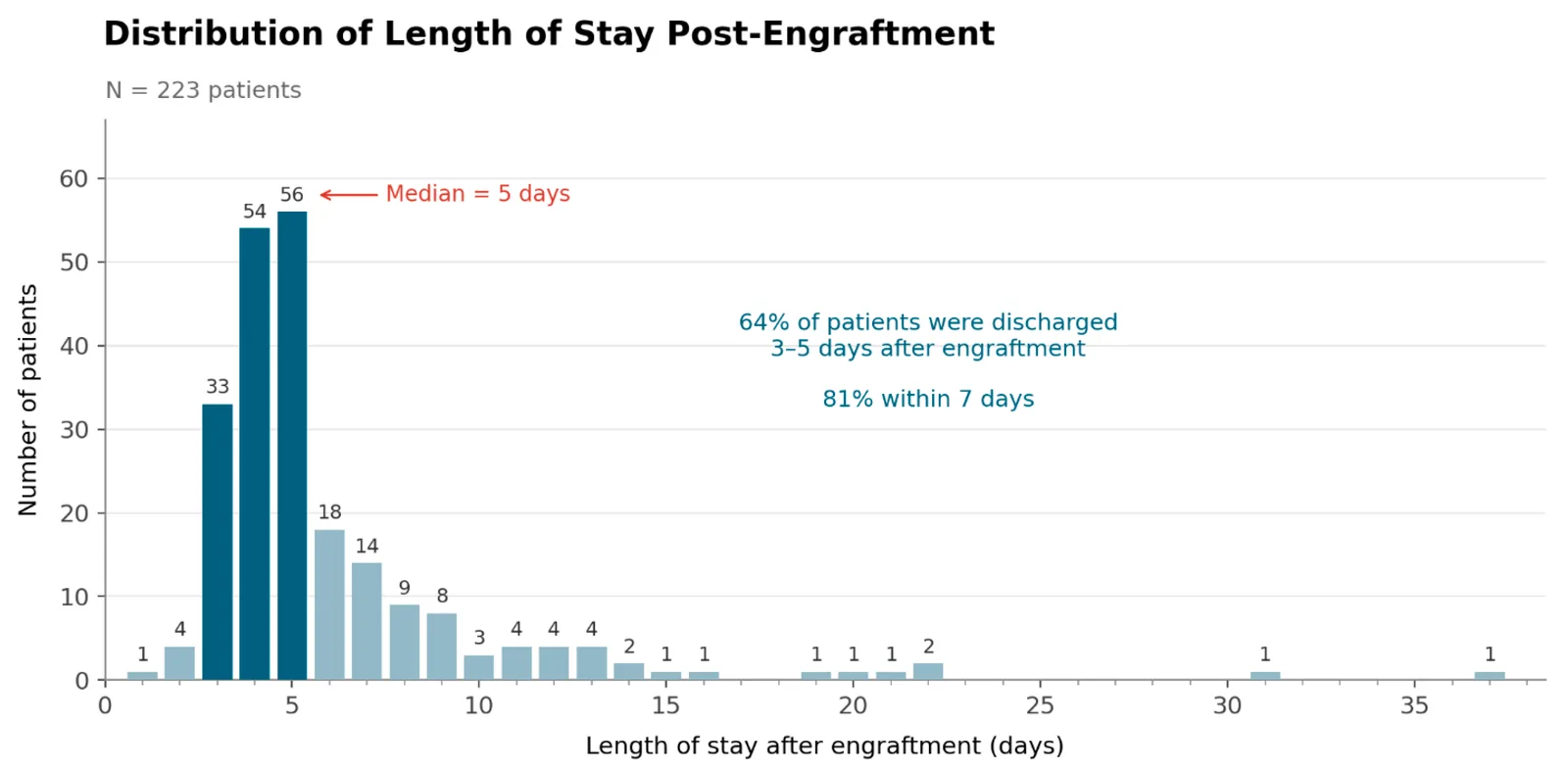
Distribution of length of hospital stay post-engraftment in days.

Figure 1 and Figure 2 present distributions of time to engraftment and hospital stay post-engraftment. As can be seen in Figure 1, the spread in the distribution of time to engraftment was quite small, with 217 cases achieving engraftment by day 12 and indicating that time to engraftment did not vary substantially across patients. In contrast, the distribution of length of stay post-engraftment was much more dispersed (see Figure 2), with the median stay of 5 days and 20% of the cases staying longer than 7 days, with a maximum length of stay of 37 days. Since the distribution of scores for LOS post-engraftment was quite skewed, we created a new binary variable that identified the 80th percentile for length of stay, which was 7 or more days post-engraftment and used it in some later analyses.

We computed Pearson correlations to determine whether pre-transplant characteristics, including the psychological symptoms, and other baseline variables in Table 1 and cognitive performance scores in Table 2 were examined for correlation with three outcomes treatment outcomes: time to engraftment, total length of stay and post-engraftment length of stay. There were no significant correlations between any of the predictors and time to engraftment. Only one pre-transplant variable, trait anxiety, was correlated with length of stay post-engraftment, r = 0.22, *p* = 0.003 and time to engraftment did not correlate with stay post-engraftment, r = −0.05, *p* = 0.45. To more closely examine this association, we used a non-linear curve fitting analysis (SPSS version 28). When stay post engraftment was predicted by STAI Trait scores, the results were significant for both linear, F(1,244) = 8.99, *p* < 0.001, R^2^ = 0.052, and non-linear, F (2,243) = 4.88, *p* = 0.005, R^2^ = 0.048, models.

**Figure 3:**
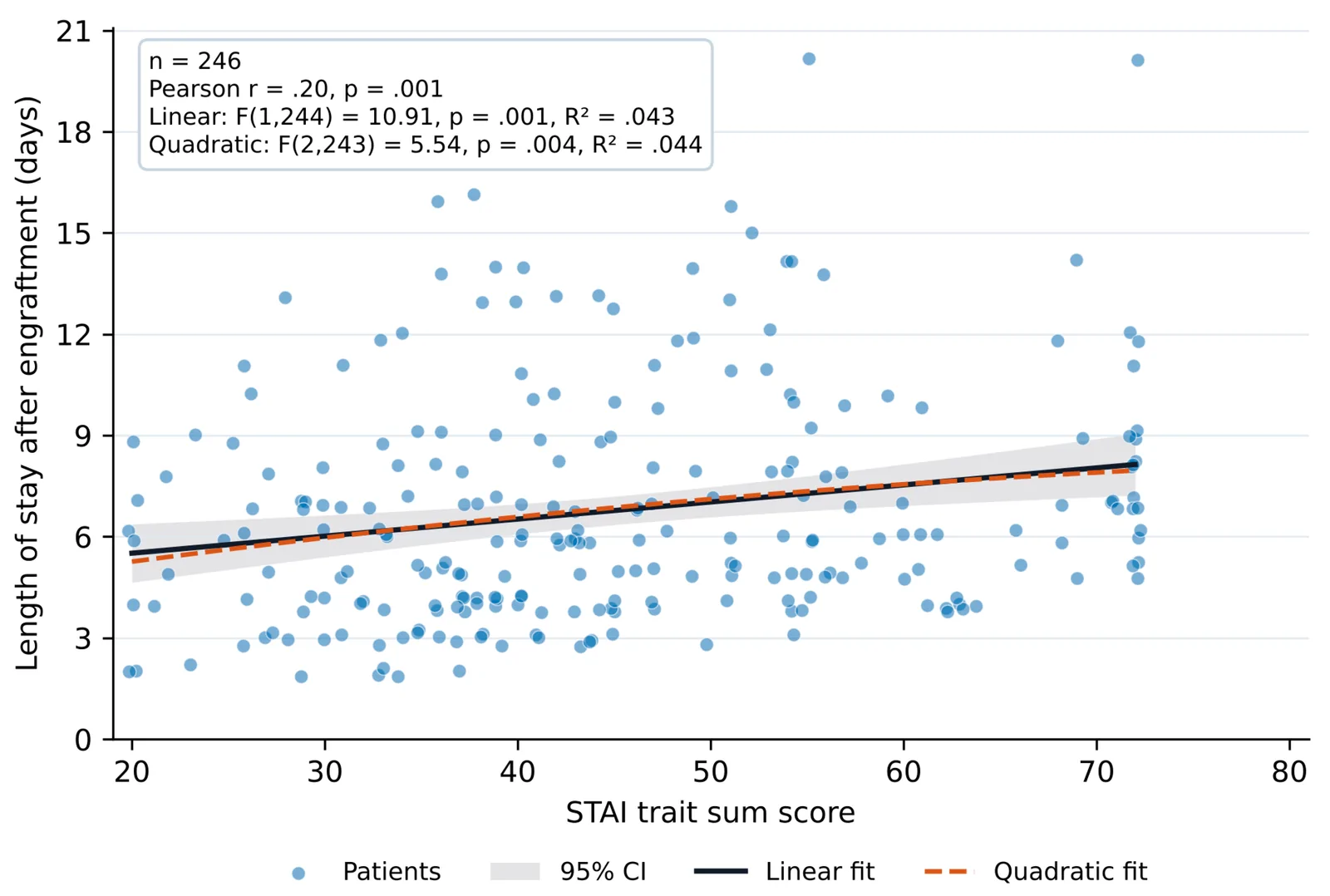
Length of Stay Post Engraftment predicted by State-Trait Anxiety Trait Anxiety score.

See Figure 3 for a scatter plot of these results.

Medical co-morbidity indexed by the HCT-CI was not correlated with any of the three variables, all r < 0.10, all, *p* > 0.39. Spanish speakers were found to have higher scores on trait anxiety, t = 2.42, *p* = 0.016. However, Spanish speakers did not have longer post-engraftment stays: m = 6.03 vs. 6.04 days for English speakers, t = 0.02, *p* = 0.99. Trait anxiety was correlated with post-transplant length of stay in the larger subsample of English speakers, r = 0.28, *p* = 0.002 and was still in the same direction in the Spanish speakers only, r = 0.16, *p* = 0.22.

- **Survival Following Transplant and Beyond**

We examined survival across four timepoints: 100 days post-transplant, one-year post-transplant, two years post-transplant, and eleven years post-transplant. All but one participant survived within the first 100 days following transplant (0.4% of the sample). This one patient died at day 29 post-transplant as a result of influenza A infection and the development of respiratory failure, multiorgan dysfunction with cardiac shock, and acute renal failure. Within one-year post-transplant, eight patients had died (3.2%) and twenty participants died within two years following transplant (8%). Within 11 years, 87 participants had died (34.8%).

The overwhelming majority of patients survived the period of highest risk for transplant-related complications (first 100 days after ASCT), resulting in very low transplant-related mortality. Rates of mortality at one-year and two-year post-transplant also remained low. Given these findings, we focused our analyses on long-term survival, during which outcomes are increasingly influenced by both disease progression and non-relapse causes, including infection, second primary malignancies, cardiovascular disease, and other treatment-related complications. We selected post-engraftment length of stay as the predictor because it represents the portion of hospitalization that extends beyond hematopoietic recovery and is therefore more likely to reflect influences of post-engraftment complications and patient-level vulnerability rather than the correlates of transplantation success.

Independent-sample *t*-tests were performed to compare patients who were alive at 11-year post-transplant (long-term survivors) and those who were deceased (non-survivors) on pre-transplant psychosocial clinical and cognitive variables. The *t*-tests revealed no significant differences between long-term survivors and non-survivors on any of the psychiatric clinical variables, all t < 1.53, all *p* > 0.13. For the cognitive variables, neither Trail Making Test Part A or B differed significantly, both t < 0.25, both *p* > 0.75. In contrast, long-term survivors demonstrated significantly better overall cognitive functioning, with higher RBANS total index scores than non-survivors. The total index scores on the RBANS were higher in long-term survivors (M = 91.35, SD = 13.40) than non-survivors (M = 87.65, SD = 14.51), t = 1.98, *p* < 0.05. Follow-up analyses of the five individual subscales were performed, finding only that delayed memory performance was significantly higher in survivors (M = 95.16, SD = 12.35) than non-survivors (M = 91.43, SD = 15.44), t = 1.99, *p* < 0.05, whereas the remaining cognitive domains did not differ significantly between groups, all t < 1.78, all *p* > 0.08. There were no differences in survival associated with HCT-CI risk, t = 0.87, *p* = 0.38. Being assessed in Spanish versus English was not associated with performance differences in either of the RBANS index scores that separated survivors and non-survivors, both t < 1.69, both *p* > 0.09. Long-term survivors also had significantly shorter total hospital stays following transplant (M = 16.04, SD = 4.05) than non-survivors (M = 17.64 days SD = 5.44), (t = 2.49, *p* = 0.013) and a there was a similar significantly shorter length of hospital stay after engraftment for survivors (M = 5.50, SD = 3.98) compared to non-survivors (M = 6.80, SD = 5.15), (t = 2.09 *p* = 0.038). There was also a significantly shorter time from diagnosis to transplant in survivors (M = 934.54 days, SD = 4844.34) compared to non-survivors, (M = 4370.87 days, SD = 11,954.26) (t = −2.95, *p* = 0.004), which may reflect differences in disease course, treatment timing, or patient selection prior to transplantation. Neither the number of prior lines of therapy (*t* = 1.81, *p* = 0.072) nor time to engraftment (*t* = 1.21, *p* = 0.226) differed significantly between survivors and non-survivors.

- **Long-term Survival Analyses**

A Cox regression model was used to identify predictors of time to mortality. The three variables that differed by long-term survivor status were entered into a forward entry model: time from diagnosis to transplant, hospital stay post-engraftment, and RBANS delayed recall scores. We chose to examine post-engraftment length of stay which more specifically reflects the period of hematopoietic recovery. We hypothesized this because the time to engraftment did not differ substantially across patients, whereas post-engraftment length of stay varied widely, indicating that non-myeloma clinical factors likely contributed to prolonged hospitalization. These factors may include treatment-related toxicities, infectious complications, delayed functional recovery, and other medical complications, making post-engraftment length of stay a clinically meaningful marker of patient vulnerability associated with long-term mortality. The results of these analyses, including all variables that were entered into the model, are presented in Table 3. The overall model was significant, *p* = 0.045. In stepwise analyses, only stay post-engraftment was a significant predictor, with time from diagnosis to treatment and delayed recall scores not entering the equation.

As a secondary analysis to enhance clinical interpretability, the Cox proportional hazard model was repeated using an empirically derived cutoff corresponding to the 80th percentile for post-engraftment length of stay (≥7 days). The overall model remained significant, *p* = 0.035. In this analysis, stays over the 80th percentile entered the equation first, *p* = 0.025, followed by RBANS delayed memory performance, *p* = 0.03, with time from diagnosis to treatment again not entering the equation.

To provide a clinically meaningful description of this association, Kaplan Meier analysis comparing patients with post-engraftment hospital stays of <7 days and ≥7 days (80th percentile) and participants with lower versus normal-range scores on delayed recall memory. Delayed recall performance was divided at an age-corrected scaled score of 85 or less (−1.0 SD compared to age-corrected normative standards <17th percentile).

Patients with prolonged post-engraftment hospitalization experienced substantially higher overall mortality (57% vs. 31%), X^2^(1) = 6.354, *p* = 0.01 and there were also differences in survival time in those who did die during the follow-up period. For lower vs. higher scores on delayed recall memory participants without impairments had a 32% risk of mortality while those with impairments had a 45% risk, which was not a significant difference, X^2^(1) = 2.25, *p* = 0.13 The results of the analyses are also presented in Table 3. Among patients who died, those with shorter post-engraftment hospital stays had a longer mean and median survival times than those with prolonged post-engraftment hospitalization, with differences in median survival times being significant defined by exceeding the 95% CI. Because the ≥7-day threshold was empirically derived from this cohort, these findings should be considered exploratory and require validation in independent cohorts. Median scores for survival were also longer for particiapnts with higher delayed recall scores, with differences in median survival times being significant, as defined by exceeding the 95% CI.

**Figure 4:**
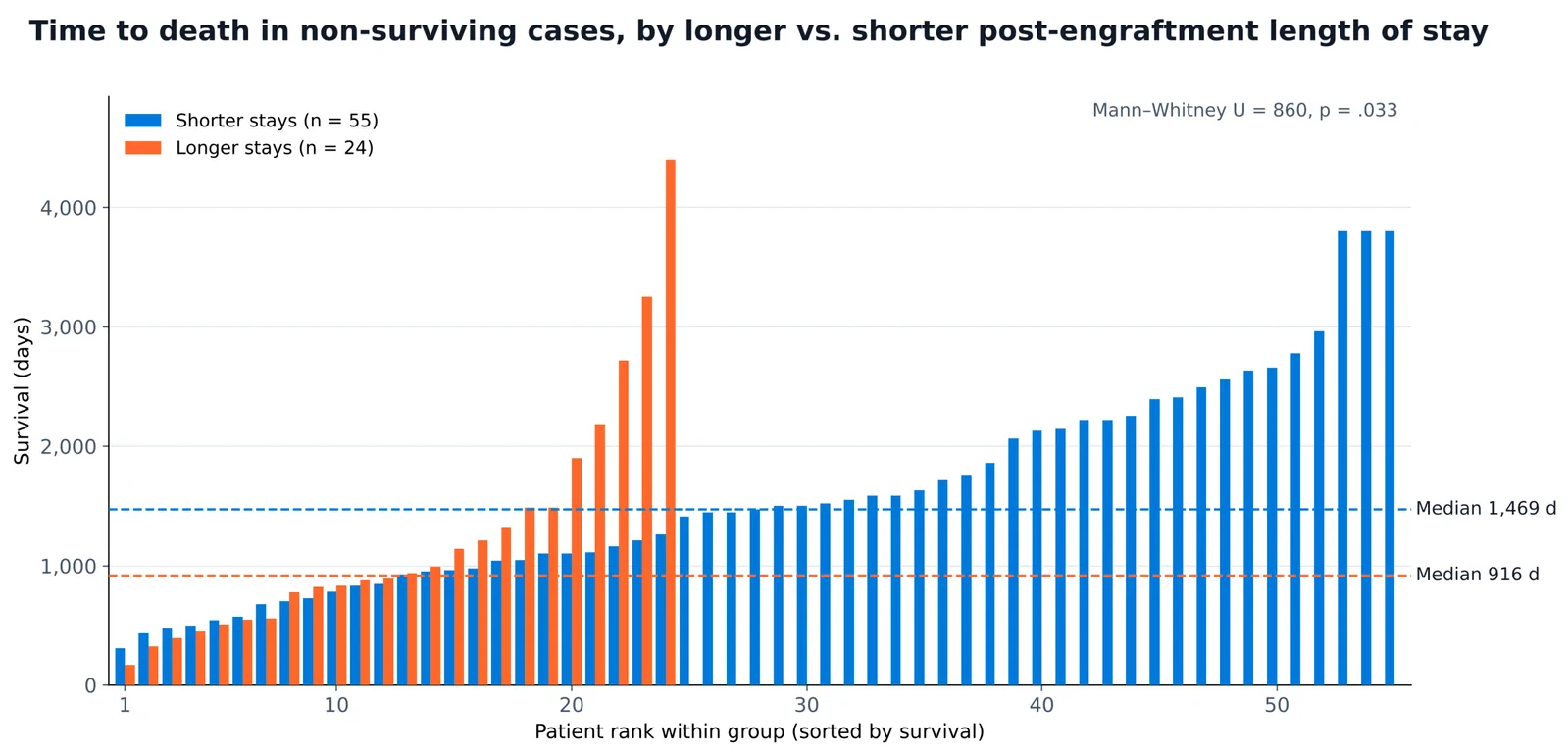
Time to death in non-surviving cases: Presented as a function of longer versus shorter length of stay.

**Figure 5:**
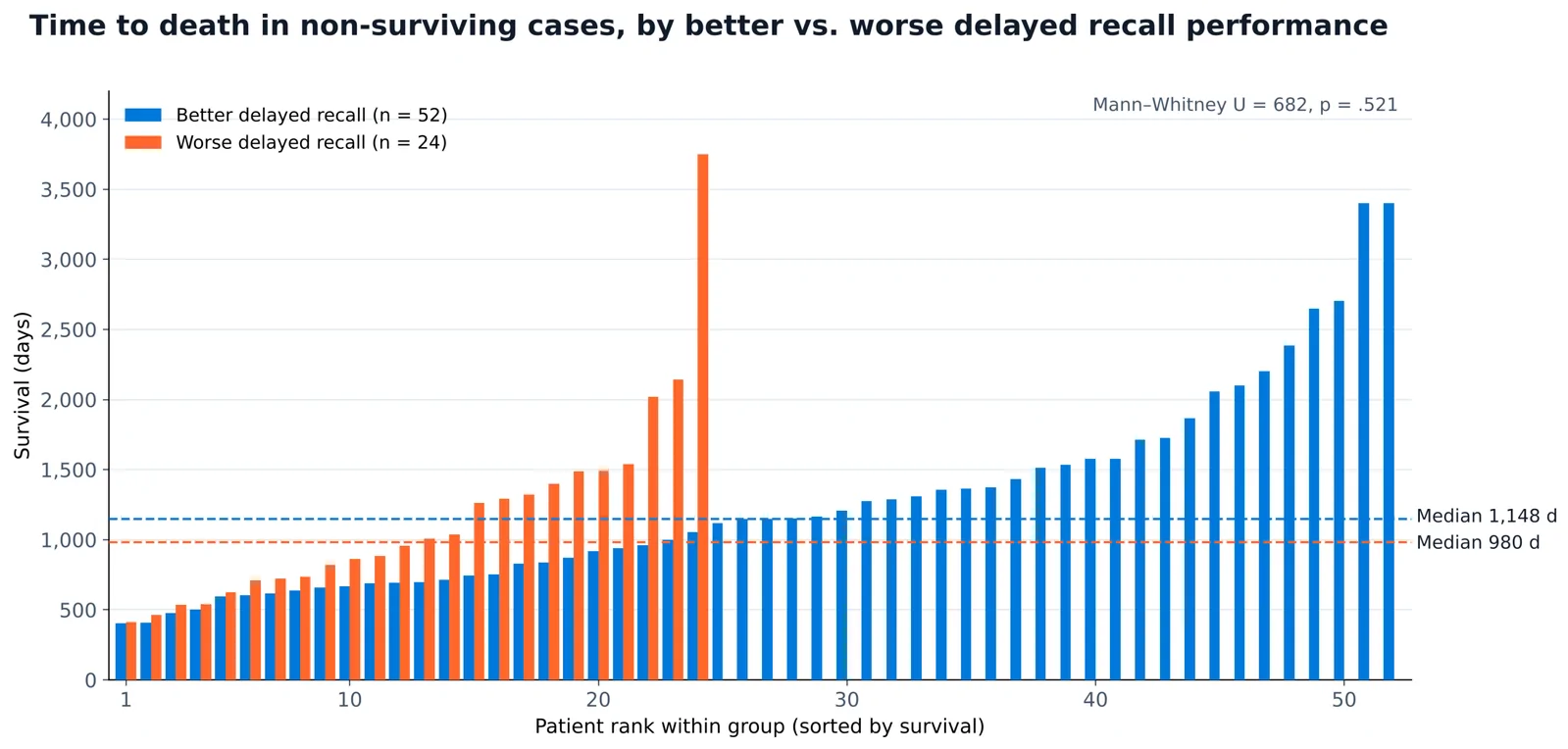
Time to death in non-surviving cases: Presented as a function of lower versus higher delayed recall performance.

Figure 4 and Figure 5 depict the results of Kaplan-Meyer life table analyses life tables examining duration of survival in non-surviving cases as a function of stays of <7 days and ≥7 days and lower versus higher delayed recall performance. Delayed recall performance was divided at an age-corrected scale score of 85 or less (−1.0 SD compared to age-corrected normative standards <17th percentile).

## 4. Discussion

In terms of the outcomes of treatment, time to engraftment and recovery post-engraftment are both potentially important variables. Time to engraftment did not vary much across patients, with most patients successfully engrafted within a very similar time frame, suggesting fairly homogenous rates of success of the procedure. In contrast, the number of days patients remained hospitalized after engraftment varied widely. Because a small group of patients had much longer hospital stays, we defined “prolonged post-engraftment hospitalization” as staying 7 or more days after engraftment (the top 20% of stays) and used this threshold in our analyses. Overall, these findings suggest that most aspects of baseline cognitive performance and most pre-transplant clinical and psychosocial characteristics were not associated with engraftment, hospital length of stay, or mortality. The only exception was trait anxiety, indicating that patients with a longstanding predisposition toward anxiety tended to experience longer hospitalizations after engraftment. The STAI instrument differentiates between transient anxiety related to current circumstances (state anxiety) and a stable disposition to experience anxiety across situations (trait anxiety). There is limited but supportive evidence that pre-transplant anxiety predicts increased length of stay specifically in the auto-SCT population. The existing literature tends to examine depression more than anxiety as a predictor of LOS, and most studies use mixed transplant populations (autologous + allogeneic) and mixed diagnoses rather than myeloma-only samples. Consistent with our findings, previous studies have demonstrated that pre-transplant psychological distress is associated with less favorable transplant outcomes. Psychiatric disorders, including mood, anxiety, and adjustment disorders, have been independently associated with longer hospital length of stay after stem cell transplantation, even after adjustment for medical risk factors [31]. Higher levels of baseline anxiety have also been linked to a poorer quality of life during hospitalization and an increased likelihood of rehospitalization following hematopoietic cell transplantation [32]. Although studies in patients with multiple myeloma undergoing ASCT have suggested that impairments in physical function and activities of daily living (ADL) are stronger predictors of prolonged hospitalization than anxiety alone, anxiety has consistently emerged as an important contributor to adverse recovery outcomes [33]. In our samples, we have begun collecting physical functioning and ADL to supplement our current results. Current transplant guidelines similarly recognize that psychological distress, particularly depression, is associated with prolonged hospitalization and increased mortality [34]. Our findings extend the literature by demonstrating that trait anxiety, which reflects a stable predisposition to perceive situations as threatening, making it a plausible predictor of how patients cope with the prolonged stress of post-engraftment recovery may lead to heightened symptom reporting, greater distress-related medical utilization, or physiological stress responses that delay discharge. We did not see a correlation with depression, but we also used only a single self-report measure to capture this syndrome. Together, these findings suggest that enduring psychological vulnerability may contribute to a more complicated post-transplant recovery and identify patients at greater risk for prolonged hospitalization.

In this cohort, the rates of survival following ASCT were in line with transplant-related mortality as reported in the literature [35]. Approximately one-third of patients died during the 11-year follow-up period, providing an opportunity to identify factors associated with long-term survival, which are not indicative of transplant-related mortality. Baseline clinical characteristics, including demographic and disease-related variables, as well as measures of processing speed and executive functioning, were not associated with mortality. In contrast, survivors demonstrated better global scores for cognitive functioning on a relatively comprehensive neuropsychological assessment before transplantation, an association driven specifically by superior delayed memory performance. This finding suggests that pre-transplant memory impairment may represent an important indicator of risk for poor outcomes in patients undergoing ASCT. This finding is consistent with prior evidence that cognitive impairment is associated with inferior outcomes, including increased toxicity and re-admissions after ASCT [36]. Pre-transplant cognitive impairment has been reported in up to 47% of patients, and memory deficits in particular have been linked to survival in patients with hematologic malignancies [37]. In our study, long-term survivors also experienced significantly shorter total and post-engraftment hospital stays, whereas neither time to engraftment nor the number of prior lines of therapy differed between survivors and non-survivors. These findings suggest that the quality of recovery following engraftment, rather than the speed of hematopoietic recovery itself, may have greater prognostic significance. In multivariable Cox regression analyses, post-engraftment length of stay remained independently associated with long-term mortality when modeled as a continuous variable, whereas baseline delayed memory performance and time from diagnosis to transplant were no longer independently associated with survival as a continuous variable. In a secondary analysis using an empirically derived cutoff corresponding to the 80th percentile of post-engraftment length of stay (≥7 days), both prolonged hospitalization and poorer baseline delayed memory were independently associated with mortality. Together, these findings suggest that prolonged post-engraftment hospitalization serves as a clinically meaningful indicator of post-transplant vulnerability and that baseline cognitive impairment may identify patients who are particularly susceptible to adverse recovery trajectories. These findings, using an assessment with extensive age norms, suggest that patients with both cognitive vulnerability and a prolonged post-transplant recovery represent a particularly high-risk subgroup which was not associated with testing language. Descriptive survival analyses further demonstrated that patients with prolonged post-engraftment hospitalization experienced higher mortality rates and shorter survival than those with shorter post-engraftment hospital stays. These findings should be interpreted as evidence of an association rather than causation and are consistent with prolonged post-engraftment hospitalization serving as a marker of greater clinical vulnerability during recovery. These results are consistent with prior work demonstrating that prolonged hospitalization and length of stay are independently associated with inferior overall survival after ASCT, and that non-hematological toxicities and infections remain important limitations for discharge even after rapid engraftment [10,18]. Collectively, these findings suggest that prolonged hospitalization following engraftment reflects greater medical complexity during the post-transplant recovery period including treatment-related toxicities, infectious complications, delayed functional recovery, and other non-myeloma factors that may influence recovery after hematopoietic reconstitution.

The observation that baseline delayed memory was associated with mortality in univariate analyses, together with its independent association in the secondary analysis using a categorical measure of prolonged post-engraftment hospitalization, supports the potential clinical value of pre-transplant cognitive assessment for identifying patients at increased risk for adverse outcomes, consistent with recent expert consensus recommendations [38,39]. However, the present study was not designed to evaluate mediation, and this hypothesis should be examined in future longitudinal studies using formal mediation analyses.

The relatively large sample size (*n* = 245) supported the stability of the multivariable analyses and allowed for the identification of independent predictors of mortality, including post-engraftment length of stay and delayed memory performance.

There are several limitations of the study. This was a purely observational study and the assessments that were administered prior to ASCT were not systematically re-administered after the transplant. Several clinical factors that may influence post-transplant outcomes were not systematically collected and could not be included in our analyses. These include disease stage, high-risk cytogenetic abnormalities, disease status and depth of response at transplant, induction regimen, performance status, and renal function. More advanced or biologically aggressive disease and more intensive prior therapy may contribute to heightened anxiety. Anemia, renal impairment, and corticosteroid exposure may adversely affect memory performance. These same factors are associated with treatment-related toxicity, delayed recovery, and mortality, so the associations we observed may be partly explained by these unmeasured clinical differences. Comorbidity burden, assessed with the HCT-CI, was not associated with any outcome. However, this index was developed to predict non-relapse mortality after allogeneic transplantation, and its performance in the autologous setting has been variable. Its predictive value here may also have been limited by the restricted range of comorbidity among patients already deemed medically eligible for transplant [40]. Similarly, the specific causes of prolonged post-engraftment hospitalization, such as infection or regimen-related toxicity, were not systematically captured. Post-engraftment length of stay should therefore be viewed as a composite marker that likely reflects, in part, clinical vulnerability not measured in this study. Treatments during the follow-up period were not systematically collected and could not be used to predict outcomes. Long-term survival in multiple myeloma is substantially influenced by disease biology and cytogenetic risk, the use or absence of maintenance therapy after ASCT, and subsequent treatments at relapse. Because patients were transplanted between 2011 and 2016, a period when maintenance practices and available salvage therapies were changing rapidly, variation in these treatments may have influenced survival independently of baseline cognitive status or post-engraftment recovery. We were also unable to distinguish relapse-related from non-relapse mortality. For these reasons, the associations with long-term mortality should be regarded as hypothesis-generating.

Prediction of post-engraftment length of stay did not reveal predictors that would suggest modifications in post-transplant in-hospital clinical treatments. Given the importance of post-engraftment LOS, this is clearly a topic for further research attention. Cognitive performance can be affected by medical and psychosocial factors, suggesting that poor memory performance may be an observable manifestation of other influences on outcome. Regardless of origin, the memory performance seen in some participants was quite poor, with 10% of the non-survivors performing at less than the age-corrected third percentile on delayed recall, (−2 SD) compared to 3% of survivors. Future studies should determine whether pre-transplant cognitive and psychological measures provide prognostic information beyond established clinical and biological risk factors.

## 5. Conclusions

In patients with multiple myeloma undergoing ASCT, baseline delayed memory impairment and prolonged post-engraftment hospitalization were associated with poorer long-term survival. Prolonged post-engraftment hospitalization remained independently associated with mortality in multivariate analyses, suggesting that it serves as a clinically meaningful marker of post-transplant vulnerability rather than a direct determinant of survival. These findings support the potential value of routine pre-transplant cognitive assessment and careful monitoring of patients after discharge who experience prolonged recovery following engraftment to identify individuals who may benefit from enhanced supportive care before, during, and after transplantation. Future studies should validate these findings in independent cohorts, determine whether interventions attempting to reduce the burden of cognitive challenges or post-transplant complications improve long-term outcomes, and formally evaluate whether post-engraftment recovery mediates the relationship between baseline cognitive function and survival.

## Figures and Tables

**Table 1:** Demographics and psychosocial variables.

Characteristic	M (SD) or *n* (%)
**Demographics**	
Age, years	58.93 (8.12); range 31–74
Sex	
Female	119 (48.6)
Male	126 (51.4)
Ethnicity	
Hispanic	118 (48.2)
Non-Hispanic	127 (51.8)
Race	
White	171 (69.8)
Black	65 (26.5)
Other/missing	9 (3.7)
**Language**	
Non-Spanish speaker	156 (63.7)
Testing language	
English	161 (65.7)
Spanish	84 (34.3)
**Treatment history**	
Lines of therapy received	
≥1 line	243 (99.2)
≥2 lines	45 (18.4)
≥3 lines	10 (4.1)
4 lines	3 (1.2)
HCT-CI score, *M* (*SD*) [range]	2.91 (2.25) [0–19]
**Psychosocial measures**	
Depressive symptoms (BDI)	8.81 (7.57)
State anxiety (STAI-S)	33.94 (12.50)
Trait anxiety (STAI-T)	31.98 (10.49)
Perceived social support (MSPSS)	6.22 (1.15)
Alcohol use (AUDIT total)	0.88 (1.43); range 0–8

Values are *n* (%) unless otherwise indicated. Percentages may not sum to 100 because of rounding. Higher scores on the BDI-II, STAI, and AUDIT indicate greater symptom severity; higher MSPSS scores indicate greater perceived social support. BDI-II = Beck Depression Inventory-II; STAI-S/STAI-T = State-Trait Anxiety Inventory, State and Trait scales; MSPSS = Multidimensional Scale of Perceived Social Support; AUDIT = Alcohol Use Disorders Identification Test; HCT-CI = Hematopoietic Cell Transplantation-Specific Comorbidity Index.

**Table 2:** Cognitive test performance and outcome variables.

Variable	M	SD	Range
**Clinical outcomes (days)**			
Length of hospital stay	16.63	4.66	—
Time to engraftment	10.66	1.59	—
Post-engraftment length of stay			
Died after transplant	6.80	5.15	1–31
Alive after transplant	5.50	3.98	2–37
**Trail Making Test (seconds)**			
Part A	38.39	17.59	—
Part B	96.63	59.16	—
**RBANS index scores**			
Immediate Memory	92.71	14.65	—
Visuospatial/Constructional	87.99	16.95	—
Language	92.52	12.61	—
Attention	97.31	18.42	—
Delayed Memory	93.73	13.71	—
Total Scale	89.89	14.04	—

Note. RBANS = Repeatable Battery for the Assessment of Neuropsychological Status. RBANS scores are age-normed standard scores (M = 100, SD = 15). Higher Trail Making Test times indicate poorer performance.

**Table 3:** Long-term survival analysis. Cox regression model.

**Panel A. Stepwise Survival Models**						
Model and predictor				χ^2^	*df*	*p*
**Model 1: Post-engraftment length of stay as a continuous predictor**						
Overall model				8.03	3	0.045
Step 1: Post-engraftment length of stay				4.49	1	0.034
Step 2: RBANS Delayed Memory				3.37	1	0.06
Step 3: Time from diagnosis to transplant				1.66	1	0.20
**Model 2: Length of stay dichotomized at the 80th percentile**						
Overall model				8.58	3	0.035
Step 1: Longer vs. shorter stay				5.04	1	0.025
Step 2: RBANS Delayed Memory				4.51	1	0.030
Step 3: Time from diagnosis to transplant				1.01	1	0.32
**Panel B. Kaplan–Meier estimates of survival time (days)**						
Post-engraftment stay	Mean	SE	95% CI	Median	*SE*	95% CI
Below 80th percentile	1461.19	110.94	[1243.76, 1678.63]	1314.00	153.52	[1013.10, 1614.90]
80th percentile or above	1254.04	205.86	[850.55, 1657.53]	952.00	51.63	[850.81, 1053.19]
Delayed Memory > 84	1441.50	113.44	[1219.17, 1663.63]	1344.00	148.31	[1022.29, 1605.71]
Delayed Memory < 85	1050.94	190.39	[677.78, 1424.11]	878.00	185.42	[514.57, 1241.43]

Note. RBANS = Repeatable Battery for the Assessment of Neuropsychological Status; CI = confidence interval. The 95% CI for each group’s median survival time excludes the other group’s median, which suggests a meaningful difference in median survival between shorter and longer post-engraftment stays.

## Data Availability

The datasets generated during and/or analyzed during the current study are available from the corresponding author on reasonable request.

## Abbreviations

The following abbreviations are used in this manuscript:

ASCT	Autologous stem cell transplantation
MM	Multiple Myeloma
CPs	Clonal Plasma cell
LOS	Length of stay
GI	Gastrointestinal
HSCT	Hematopoietic stem cell transplantation
